# How can gastro-intestinal tuberculosis diagnosis be improved? A prospective cohort study

**DOI:** 10.1186/s12879-020-04983-y

**Published:** 2020-03-30

**Authors:** Christopher Lowbridge, Soraya A. M. Fadhil, Gayathri D. Krishnan, Emma Schimann, Raman Muthu Karuppan, Nagaraj Sriram, Giri Shan Rajahram, Jayaram Menon, Aatish Patel, Timothy William, Dawn Carmel Paul, Anna P. Ralph

**Affiliations:** 1 Global and Tropical Health Division, Menzies School of Health Research, Charles Darwin University, Box 41096, Casuarina NT, Darwin, PO 0811 Australia; 2 Department of Medicine, Queen Elizabeth Hospital, Kota Kinabalu, Malaysia; 3 Infectious Diseases Society Sabah-Menzies School of Health Research Clinical Research Unit, Kota Kinabalu, Sabah Malaysia; 4 Jesselton Medical Centre, Kota Kinabalu, Malaysia; 5 Public Health Laboratory (Makmal Kesihatan Awam), Kota Kinabalu, Malaysia

**Keywords:** Gastro-intestinal tuberculosis, Extra-pulmonary tuberculosis, Cohort study, Diagnosis, GeneXpert

## Abstract

**Background:**

Gastrointestinal tuberculosis (TB) is diagnostically challenging; therefore, many cases are treated presumptively. We aimed to describe features and outcomes of gastrointestinal TB, determine whether a clinical algorithm could distinguish TB from non-TB diagnoses, and calculate accuracy of diagnostic tests.

**Methods:**

We conducted a prospective cohort study of hospitalized patients in Kota Kinabalu, Malaysia, with suspected gastrointestinal TB. We recorded clinical and laboratory characteristics and outcomes. Tissue samples were submitted for histology, microscopy, culture and GeneXpert MTB/RIF®. Patients were followed for up to 2 years.

**Results:**

Among 88 patients with suspected gastrointestinal TB, 69 were included in analyses; 52 (75%) had a final diagnosis of gastrointestinal TB; 17 had a non-TB diagnosis. People with TB were younger (42.7 versus 61.5 years, *p* = 0.01) and more likely to have weight loss (91% versus 64%, *p* = 0.03). An algorithm using age < 44, weight loss, cough, fever, no vomiting, albumin > 26 g/L, platelets > 340 × 10^9^/L and immunocompromise had good specificity (96.2%) in predicting TB, but very poor sensitivity (16.0%). GeneXpert® performed very well on gastrointestinal biopsies (sensitivity 95.7% versus 35.0% for culture against a gold standard composite case definition of confirmed TB). Most patients (79%) successfully completed treatment and no treatment failure occurred, however adverse events (21%) and mortality (13%) among TB cases were high. We found no evidence that 6 months of treatment was inferior to longer courses.

**Conclusions:**

The prospective design provides important insights for clinicians managing gastrointestinal TB. We recommend wider implementation of high-performing diagnostic tests such as GeneXpert® on extra-pulmonary samples.

## Background

Gastrointestinal tuberculosis (TB) is a relatively uncommon form of TB which is defined as infection of the peritoneum, abdominal organs or abdominal lymphatic system [1]. TB control programs typically focus on pulmonary TB which is the predominant form of the disease; extra-pulmonary TB, in particular gastro-intestinal TB, is relatively neglected. This is likely due to it being more difficult to diagnose and lacking the transmission potential of smear-positive pulmonary TB. However, extra-pulmonary TB, including gastrointestinal TB often represents the worst end of the TB disease severity spectrum, with poorer prognosis and treatment outcomes [2]. Severe and potentially life-threatening complications of gastrointestinal TB include intestinal strictures, obstruction, perforation and bleeding [3].

TB is a disease of major public health importance in Malaysia. Sabah State, located in Malaysian Borneo, has a particularly high burden of disease, accounting for approximately 20 to 30% of Malaysia’s TB cases despite having only 10% of Malaysia’s population [4, 5].

Gastrointestinal TB is particularly difficult to diagnose, with only a minority of cases able to be microbiologically confirmed. This creates risks of both under and over-diagnosis, uncertainty about best clinical approaches, and potential under-reporting of cases to the national TB register. These diagnostic difficulties in Sabah are compounded by the presence of important differential diagnoses which can mimic the various manifestations of gastrointestinal TB, including infectious causes (e.g. *Burkholderia pseudomallei*), [6] and non-infectious causes (inflammatory bowel disease, lymphoma other malignancies).

This study describes the presentation, diagnosis, management and short and long-term outcomes of gastrointestinal TB cases at the Queen Elizabeth Hospital and Women’s and Children’s Hospital in Sabah, Malaysia. Through analysis of presenting signs and symptoms, clinical evaluation and diagnostic results, we aimed to develop a clinical algorithm to aid in the diagnosis of gastrointestinal TB. A clinical algorithm or score could help categorize patients as having gastrointestinal TB or an alternative diagnosis while awaiting, or in the absence of, further diagnostic information. Further we estimated the sensitivity and specificity of diagnostic tests, including GeneXpert® for laboratory confirmation of gastrointestinal TB.

## Methods

### Study setting and design

We conducted a prospective, observational study of the presentation, diagnosis, management and outcomes of gastrointestinal TB cases presenting to Queen Elizabeth Adult Hospital, Kota Kinabalu, Sabah, Malaysia. Sequential patients presenting to Queen Elizabeth Hospital with suspected gastrointestinal TB and referred to the Departments of Medicine, Surgery, Gastroenterology or Infectious Diseases were eligible for inclusion. Diagnostic work-up of patients presenting with suspected gastrointestinal TB included blood, microbiological, histopathological and other tests (see supplementary material Table 1). Patients were classified as ‘confirmed’, ‘probable’, or ‘possible gastrointestinal TB’, or as ‘not TB’ using standard case definitions (see supplementary material Table 2).

Samples were processed at the research laboratory at Queen Elizabeth Hospital (GeneXpert MTB/RIF®), the pathology department of Queen Elizabeth Hospital (histology) and the Public Health Laboratory of Kota Kinabalu, Malaysia (culture, TB polymerase chain reaction [PCR]). Mycobacterial culture was performed using Ogawa media. PCR was performed using the Seegene™ Anyplex MTB/NTM kit (estimated sensitivity versus culture: 100%) [7]. To prepare tissue samples for GeneXpert testing, samples were handled in a biosafety cabinet and were homogenized using a sterile mortar and pestle or chopped / mashed finely in a sterile petri dish with a scalpel blade. Approximately 1 mL sterile saline was added. A sterile disposable pipette was used to transfer the material to a tube for mixing and incubation with the GeneXpert buffer (1 mL sample to 2 mL buffer). After 15 min of mixing and incubation, a sterile disposable pipette was used to transfer only liquid (no particulate matter) into the MTB/RIF cartridge.

Participants with definite, probable or possible gastrointestinal TB that was proven or assumed to be fully drug susceptible were treated using a standard regimen consisting of isoniazid, rifampicin, ethambutol, and pyrazinamide for a duration of at least 6 months, in accordance with World Health Organization (WHO) and Malaysian Department of Health guidelines [8]. Divergence from the standard regimen was made at the discretion of the treating physician, due to factors such as deranged liver function tests at baseline or intolerance to one or more of the standard first-line medications.

Participants with confirmed or suspected drug-resistant TB received a regimen and treatment duration that was consistent with WHO guidelines for drug-resistant TB, which includes the recommendation for continuation of treatment for 18-months after culture conversion [9].

Follow-up of patients with gastrointestinal TB was conducted in line with standard clinical practice at the study sites. This consisted of monthly follow-up for the first 6 months, followed by follow-up every three to four months up to two years post treatment, to monitor for relapse of TB.

A standardized data sheet was used to collect demographic and epidemiological information, clinical history, vital, biochemical and microbiological data, treatment, outcome, and final diagnosis. Data were entered into a REDCap® electronic data capture tool [10] hosted at Menzies School of Health Research and analyzed using STATA® version 15. Two levels of analysis were conducted, the first comparing confirmed and probable gastrointestinal TB against patients classified as non-TB, the second level limited to comparing confirmed TB against non-TB. Outcomes of a six-month treatment regimen were assessed by determining adverse events, requirement to change treatment regimen, short-term (end of treatment) outcome and two-year relapse rates.

### Statistical analyses

Pearson’s χ2 test was used to assess the difference in distribution of categorical variables between groups. Welch’s unequal variances t-test or Kruskal-Wallis equality of populations rank test were used for analysis of difference in means of continuous variables between groups. Sensitivity and specificity of *Mycobacterium tuberculosis* culture, polymerase chain reaction (PCR) and GeneXpert MTB/RIF® as well as histology for detecting presence of *M. tuberculosis* were calculated using the confirmed TB case definition as gold standard. Histology results were blindly reviewed by three infectious diseases physicians and classified as ‘highly suggestive of TB’, ‘suggestive of TB’, ‘not suggestive of TB’ or ‘clear alternative diagnosis’. Disagreements were resolved by discussion until consensus was reached.

To develop a clinical algorithm to support diagnosis of gastrointestinal TB, we calculated the odds ratios and log odds of relevant variables (age, symptomatology, duration of symptoms, liver function tests, blood chemistry and blood count), comparing probable and confirmed TB cases to confirmed non-TB cases. We excluded variables where the *P*-value of the odds ratio was ≥0.5 and limited variables for inclusion in the model to the top eight variables based on highest absolute log odds value. Points were allocated to each of the variables in the algorithm based on log odds value, and area under the curve of receiver operator characteristics was calculated to assess sensitivity, specificity and positive predictive value of the algorithm, using final diagnosis of TB as the gold standard.

## Results

### Patient characteristics

From a total of 88 patients suspected of having gastrointestinal TB on presentation at Queen Elizabeth Hospital and the Women’s and Children’s Hospitals, Kota Kinabalu, Sabah, between March 2015 and June 2017, consent for inclusion in the study was obtained from 77 patients. Of these, eight patients were excluded from the analysis due to incomplete follow-up and final diagnosis. Among the 69 patients included in the study: 52 (75%) had a final diagnosis of gastrointestinal TB (25 confirmed, 13 probable, 14 possible cases), and 17 patients were assessed to have non-TB diagnoses, the majority of which (*n* = 12) were malignancy (Supplementary Fig. 1). 

**Fig. 1:**
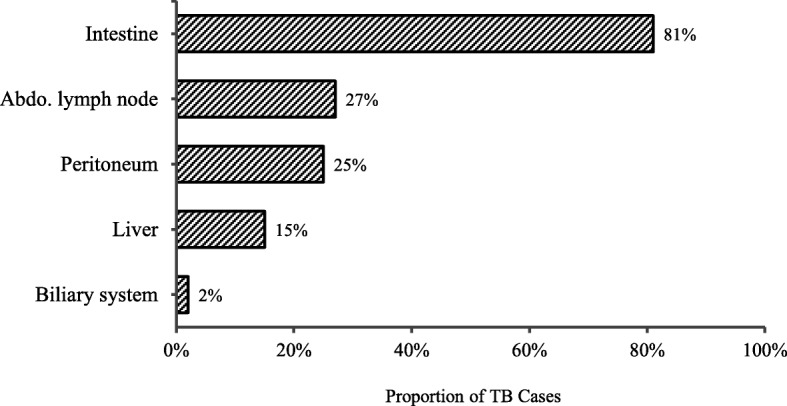
Site of gastrointestinal TB disease

Among the 52 cases of confirmed, probable and possible gastrointestinal TB, 42 (81%) had infection of the intestine, 14 (27%) had infection of an abdominal lymph node, 13 (25%) had infection of the peritoneum, 8 (15%) had infection of the liver, and 1 (2%) had biliary infection (Fig. 1). A total of 45 (87%) gastrointestinal TB cases were recorded as having involvement of multiple sites or organs within the gastrointestinal system.

**Fig. 2:**
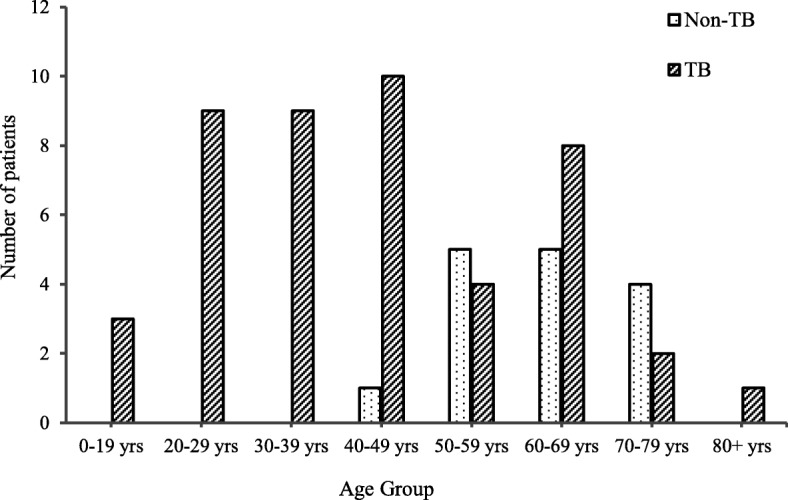
Age distribution of patients by final diagnosis

The mean age of patients with a final diagnosis of gastrointestinal TB (confirmed, probable, possible) was 42.7 years, significantly lower when compared with the mean age of 61.5 years for patients with non-TB diagnosis (*t* (46 *d.f.*) = 5.07, *p* < 0.01) (Fig. 2). There was no significant difference in sex, with 51% of gastrointestinal TB cases being male, compared with 60% of non-TB cases.

### Microbiological and histological results

A total of 110 biopsy samples were collected from patients during initial work-up and ongoing clinical follow-up. The largest proportion of samples were collected from the large bowel (34%), followed by the ileum (14%) and peritoneum (10%). Samples were also collected from the stomach, small bowel, liver and omentum. Peritoneal biopsy samples were collected predominantly via laparoscopy (*n* = 7) or laparotomy (*n* = 2).

Twenty-four of 42 TB cases tested by GeneXpert® were positive for *M.tuberculosis*. Of 39 TB cases with mycobacterial culture results, 12 had *M.tuberculosis* detected by culture. GeneXpert® positivity by sample type is detailed in supplementary Table 4. Rifampicin resistance was detected by GeneXpert® in three cases, of which one was found to be sensitive to rifampicin and another was confirmed rifampicin resistant by phenotypic drug-susceptibility testing. The third was treated as rifampicin resistant based on the GeneXpert® result. Two cases were found to be isoniazid mono-resistant by phenotypic drug-susceptibility testing.

Sensitivity and specificity of diagnostic tests for *M.tuberculosis* are presented in Table 1. GeneXpert®, culture, PCR and smear microscopy for Acid Fast Bacilli all achieved 100% specificity against the gold standard of final case classification of confirmed TB. GeneXpert® achieved high sensitivity (95.7%), compared with very low sensitivity of culture (35.0%), PCR (50.0%) and microscopy (31.0%). Histology achieved moderate sensitivity (68.0%) and specificity (77.1%) when using the lower threshold for evidence of TB (‘suggestive’ or ‘highly suggestive’ of TB).

**Table 1:** Sensitivity and specificity of diagnostic tests using gastrointestinal biopsy specimens

	***N*** tests	Sensitivity %	Specificity %	PPV %	NPV %
GeneXpert®	57	95.7	100	100	97.1
Culture	42	35.0	100	100	62.9
PCR	29	50.0	100	100	68.2
AFB	55	31.0	100	100	56.5
Histology ^a^	60	68.0	77.1	68.0	77.1
Histology ^b^	60	36.0	85.7	64.3	65.2

^a^ Histology results classified as ‘suggestive’ or ‘highly suggestive of TB’ versus results classified as not ‘suggestive of TB’ or ‘clear alternative diagnosis’

^b^ Histology results classified as ‘highly suggestive of TB’ versus results classified as ‘clear alternative diagnosis’

### Features distinguishing TB from non-TB and development of a clinical score

**Fig. 3:**
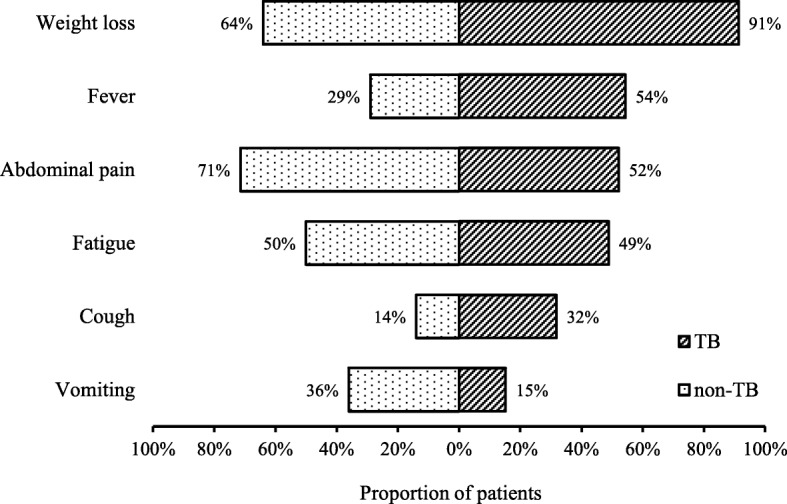
Reported symptoms at hospital admission, by TB status

Among confirmed and probable TB cases, the most common symptoms reported at presentation were weight loss (91%), fever (54%) and abdominal pain (52%). For non-TB cases, the most common presenting symptom was abdominal pain (71%), followed by weight loss (64%) and vomiting (36%) (Fig. 3). Confirmed and probable TB cases were significantly more likely to present with weight loss compared with non-TB cases (χ2 = 4.93, *p* = 0.03). The proportion of probable and confirmed TB cases with a recorded temperature of 38 degrees Celsius or higher on presentation was higher (24%) than non-TB cases (14%), however the difference was not statistically significant. Confirmed and probable TB cases presented with longer median duration of fever (4 weeks vs. 1 week) and abdominal pain (8 vs. 4 weeks) but shorter duration of weight loss (10 vs. 14 weeks), vomiting (3 vs. 18 weeks), cough (8 vs. 53 weeks) and fatigue (8 vs. 12 weeks), compared with non-TB cases. However, none of the differences in duration of symptoms between TB and non-TB cases were statistically significant.

Two patients in our study cohort had HIV infection, both with confirmed gastrointestinal TB. There were no statistically significant differences in the proportion of patients who had: a history of Bacillus Calmette–Guérin (BCG) vaccination; past TB diagnosis; family history of TB; positive tuberculin skin test; HIV infection; diabetes; or immunocompromising condition, between confirmed TB cases and non-TB cases or confirmed and probable TB cases and non-TB cases. No significant risk factors emerged when limiting the analysis to either confirmed cases of TB or non-TB cases with malignancy.

There were no statistically significant differences in blood chemistry, blood count or liver function tests between confirmed and probable TB cases and non-TB cases (supplementary material, Table 3). Non-TB cases had lower mean albumin levels (21.54 vs. 26.17 g/L) and higher mean bilirubin levels (24.0 vs. 13.35 mmol/L) from peripheral blood samples on initial diagnostic work-up—however these differences were not significant. No statistically significant differences emerged when limiting the analysis to confirmed TB cases or non-TB malignancy. Blood cultures were positive in four of four non-TB cases who had blood cultures collected (isolates were: *Elizabethkingia meningoseptica*, *Escherichia coli*, and two with *Klebsiella pneumoniae*) and one probable TB case (*Acinetobacter baumannii*).

A composite clinical score was developed to determine whether a scoring system could reliably distinguish TB from non-TB diagnosis (Table 2). The best performing algorithm for diagnosis of gastrointestinal TB included: age less than 44 years; weight loss; cough; fever; no vomiting; blood albumin level great than 26 g/L; platelet count greater than 340 × 10^9^/L; and history of immunocompromising illness/treatment. However, this algorithm achieved an area under the receiver operator characteristics curve of just 0.5873. The highest correct classification of cases (70.1%) occurred at a cut-point of eight points from a maximum of 11 points, this corresponded to a specificity of 96.2%, but sensitivity of only 16.0% (positive predictive value = 60.0%; negative predictive value = 3.8%). There was no significant difference in performance of the composite clinical score when assessed against an alternative gold standard, such as microbiologically confirmed *M.tuberculosis*.

**Table 2:** Clinical score tested for diagnosis of gastrointestinal tuberculosis

Criteria	Points
Age less than 44 years	3
Weight loss	2
No vomiting	1
Albumin > 26 g/L	1
Platelets > 340 × 10^9^/L	1
Cough	1
Immunocompromised	1
Fever > 38 °C	1
Total possible score	11

### Treatment

Details of the prescribed anti-tuberculous treatment regimen was documented for 38 patients. Standard first-line treatment of isoniazid, rifampicin, ethambutol and pyrazinamide was initiated in 32 (84%) TB cases with documentation of treatment, with proposed treatment durations at initiation of, six-months, nine-months and 12-months for 9 (28%), 16 (50%) and 7 (22%) cases respectively. Most patients (91%) received pyridoxine (vitamin B6) during treatment. Six patients were given an alternative regimen: one due to adverse reaction to ethambutol, one due to baseline liver impairment, two due to detection of rifampicin resistance and two for an undocumented reason. Of the two cases with rifampicin resistance: one was treated with a regimen consisting of kanamycin, ethionamide, cycloserine and levofloxacin; the other with a regimen consisting of isoniazid, ethambutol, pyrazinamide, kanamycin, levofloxacin and ethionamide. Mean treatment duration at completion for cases receiving first-line regimens was 8.8 months (median 9.0 months, maximum 12.4 months).

A total of 12 adverse events were reported among eight patients undergoing anti-tuberculous treatment. The majority (67%) of adverse events occurred during the first 2 months of treatment. Hepatotoxicity and rash were the most frequent adverse events reported, accounting for 25% of adverse events each. Most adverse events were mild or moderate (75%), three required hospitalization but none were life-threatening or resulting in death. Changes in treatment regimen were required in six (16%) of the 38 patients for whom treatment details were available. Five adverse events resulted in permanent cessation of a drug, two resulted in a drug being stopped and then reintroduced and one resulted in a change in route of administration of a drug. No new agents were added to any regimens because of an adverse event.

TB cases were followed-up for a median of 7.1 months (maximum 16.9 months). Final outcome was recorded for 92% of gastrointestinal TB cases. Of these, 79% successfully completed treatment or were cured; no case of treatment failure was recorded. A total of six (13%) TB cases died. TB cases who died had a mean age of 54 years and all deaths occurred in the first 5 months following hospitalization. Four cases (8%) defaulted from treatment. Three (18%) non-TB cases died (two with malignancy and one with *Helicobacter pylori* gastritis).

## Discussion

In this two-year prospective study, we found that 75% of people presenting with suspected gastrointestinal TB had a final diagnosis of TB. A composite clinical score using patient characteristics and investigation findings had good specificity in predicting a diagnosis of TB, but poor sensitivity. This highlights the importance of access to reliable diagnostic tests for diagnosing extra-pulmonary TB. We identified that GeneXpert MTB/RIF performed very well on tissue samples and was considerably superior to culture in this specific setting. This reflects a pragmatic real-world situation rather than a laboratory based head-to-head comparison of diagnostic modalities, taking into account challenges in reporting results back to treating clinicians. Other key findings from this cohort were the relatively high rate of adverse treatment events necessitating regimen changes in 16% of individuals, and the high mortality rate among TB cases of 13%. The unique study design using prospective enrolment of suspected TB cases provides important new insights for clinicians managing this condition especially with regards to diagnostic and prognostic features. The findings highlight the ongoing morbidity and mortality burden posed by TB in this endemic area.

It is well established that gastrointestinal TB is diagnostically challenging due to cases commonly presenting with non-specific symptoms, laboratory, and radiological findings [11, 12]. This is especially the case in a setting such as Sabah where numerous infections which may present similarly to TB are endemic, in addition to common non-infectious differential diagnoses of gastrointestinal TB, such as malignancy and autoimmune conditions. Delayed diagnosis can result in increased risk of complications or worse patient outcomes [13]. Findings from our prospective observational study highlight the challenge of diagnosing gastrointestinal TB. We found substantial similarities in demographic and risk factor profiles, clinical presentations and basic laboratory findings during initial assessment and work-up.

A robust algorithm for classifying gastrointestinal TB cases, as is available for TB meningitis, [14] based on scoring of clinical information would potentially improve the early diagnosis of gastrointestinal TB cases. We attempted to develop such an algorithm, based on the constellation of signs and symptoms listed in Table 2. While a high score using this algorithm was suggestive of gastrointestinal TB, its poor sensitivity and predictive values make it inadequate for use as a clinical or research classification tool.

Given the inherent challenges in clinical diagnosis and classification of gastrointestinal TB, the use of rapid diagnostics is important. The GeneXpert® MTB/RIF assay has proved a hugely useful test in the diagnosis of pulmonary TB. In the diagnosis of extra-pulmonary TB, it has been shown to be specific, however concerns have been raised about its sub-optimal sensitivity [15]. We found that GeneXpert® performed well on biopsy samples, being both highly sensitive and specific. This is in keeping with a study by Hilleman et al., which found high sensitivity and specificity of GeneXpert® MTB assay from gastric fluid and stool specimens (87.5% & 100, and 100% and 98.6% respectively) in cases of extra-pulmonary TB, [16] and a metanalysis of GeneXpert® on extra-pulmonary samples which found a pooled sensitivity and specificity of 86 and 98% respectively from gastrointestinal samples [15]. In contrast to these findings however, two studies from India, assessing the performance of GeneXpert® on intestinal biopsies found low sensitivity of 8.1 and 32% respectively [17, 18].

Caution should be applied in comparing our findings of sensitivity and specificity with other estimates, given our use of final clinical diagnosis as opposed to *M. tuberculosis* culture as the gold standard, as well as the relatively small sample sizes of published studies reporting sensitivity and specificity of GeneXpert® from gastrointestinal samples. The superiority of GeneXpert® over culture observed here may not be generalizable to settings with advanced expertise in *M. tuberculosis* culture (including recovery from contaminated samples) which may result in greater concordance between GeneXpert® and culture results. Ogawa culture medium used at the reference laboratory may have slightly lower sensitivity for *M. tuberculosis* detection than liquid culture media or other egg-based solid media such as Lowenstein-Jensen [19, 20]. Also, the laboratory lacked an electronic reporting system at the time of the study – since rectified – resulting in missing culture results in four instances where a GeneXpert® was available and positive.

Our findings emphasize the need for further investment in research and development of new tools to improve the diagnosis of gastrointestinal TB, especially the identification of novel systemic biomarkers for TB infection and disease leading to the availability of new in vitro assays.

The chief limitation of the study is the sample size. While the original cohort was relatively large for a study of gastrointestinal TB, not all people within the cohort were able to be included in the analysis, and as the majority of the cohort (75%) had a final diagnosis of TB, the number of non-TB cases was small. The sample size calculation for a descriptive study of a dichotomous variable (the dichotomous variable being diagnosis of TB or not among presenting patients) would indicate that 288 patients would be needed to be able to make estimates with 95% confidence (α = 0.05) if the proportion found to have TB was 75% [21]. The superiority of GeneXpert MTB/RIF over culture may not be reproducible in other international settings where culture facilities may be better supported. However, the reality is that high TB-burden settings often lack high-quality, accredited TB culture facilities; therefore, the findings are relevant to such settings.

We did not identify any cases of treatment failure within our study cohort. While 13% of TB cases in our cohort died, all deaths occurred less than 6 months after starting treatment. While our study was not powered to compare treatment regimens, we found no evidence that 6 months of treatment was less effective than a longer treatment course. This is consistent with a review by the Cochrane group which, although based on small numbers of patients, found no evidence that 6 months treatment duration was inadequate for treatment of gastrointestinal TB [22].

## Conclusions

Gastrointestinal TB remains diagnostically challenging. An algorithm developed to classify patients presenting with suspected gastrointestinal TB cases was specific but insensitive. Testing of such algorithms in larger populations of patients with suspected gastrointestinal TB will reveal whether a composite clinical score could help categorize patients while awaiting, or in the absence of, further diagnostic information. Wider implementation of existing high-performing diagnostic tests such as GeneXpert® on extra-pulmonary samples, and ongoing investment in new diagnostics development, is needed. We recommend increased access to and uptake of GeneXpert® on gastrointestinal samples in suspected TB cases.

## Supplementary information

**Additional file 1: Table S1.** Diagnostic work-up of patients with suspected gastrointestinal tuberculosis. **Table S2.** Gastrointestinal tuberculosis case definitions. **Figure S1.** Recruitment and classification of study participants. **Table S3.** Mean blood test results during initial diagnostic work-up. **Table S4.** Gastrointestinal biopsy and ascitic fluid GeneXpert® MTB results by type and site.

## Data Availability

The dataset generated and analyzed during the current study is not publicly available due to it containing identifying medical information. Condensed anonymized data are available from the corresponding author on reasonable request.

## Abbreviations

BCG: Bacillus Calmette–Guérin vaccine
HREC: Human Research Ethics Committee
MTB: *Mycobacterium tuberculosis*
NMRR: National Medical Research Register (Malaysia)
PCR: Polymerase chain reaction
RIF: Rifampicin
TB: Tuberculosis
WHO: World Health Organization
