# Supplementary material for: How can gastro-intestinal tuberculosis diagnosis be improved? A prospective cohort study
Source: BMC Infect Dis. 2020 Mar 30;20:255. doi: 10.1186/s12879-020-04983-y (PMC7106693; doi:10.1186/s12879-020-04983-y)
Supplement: Supplementary file 1 — Additional file 1: Table S1. Diagnostic work-up of patients with suspected gastrointestinal tuberculosis. Table S2. Gastrointestinal tuberculosis case definitions. Figure S1. Recruitment and classification of study participants. Table S3. Mean blood test results during initial diagnostic work-up. Table S4. Gastrointestinal biopsy and ascitic fluid GeneXpert® MTB results by type and site. [file 12879_2020_4983_MOESM1_ESM.docx]

# How can gastro-intestinal tuberculosis diagnosis be improved? A prospective cohort study

## Supplementary Material

## Figure Legend

Table 1: Diagnostic work-up of patients with suspected gastrointestinal tuberculosis

Table 2: Gastrointestinal tuberculosis case definitions

Figure 1: Recruitment and classification of study participants

Table 3: Mean blood test results during initial diagnostic work-up

Table 4: Gastrointestinal biopsy and ascitic fluid GeneXpert® MTB results by type and site

**Supplementary table 1: Diagnostic work-up of patients with suspected gastrointestinal tuberculosis**

| Blood tests | - Full blood count - Serum electrolytes - Liver function tests - Blood culture - HIV rapid test or serology |
| --- | --- |
| Imaging | - Chest x-ray - Abdominal x-ray, ultrasound, CT, and/or MRI as indicated |
| Microbiological testing* | - Mycobacterial microscopy - Mycobacterial culture and susceptibility testing - *M. tuberculosis* Polymerase Chain Reaction (PCR) - GeneXpert MTB/RIF® |
| Histopathology | - Standard processing for detection of granulomata, caseous necrosis etc. - Auramine staining for presence of Acid-Fast Bacilli |
| Other | - Ascitic, pleural and or pericardial fluid biochemical analysis and cell count - Tuberculin skin test |

*Diagnostic samples may include sputum, ascitic fluid, biopsies, lymph node aspirates, etc.

**Supplementary table 2: Gastrointestinal tuberculosis case definitions**

| **Confirmed** | **Probable** | **Possible** | **Not tuberculosis** |
| --- | --- | --- | --- |
| Signs, symptoms and or imaging suggesting gastrointestinal TB | Signs, symptoms and or imaging suggesting gastrointestinal TB | Signs, symptoms and or imaging suggesting gastrointestinal TB | Signs, symptoms and or imaging suggesting gastrointestinal TB |
| **and** | **and** | **and** | **but** |
| *M. tuberculosis* culture positive on gastrointestinal specimen | Acid Fast Bacilli detected on microscopy on gastrointestinal specimen | Supportive findings e.g. mononuclear predominance in ascitic fluid, ileal inflammation on endoscopy | Alternative diagnosis confirmed or strongly suspected (e.g. alternative pathogen identified, or illness resolves quickly without TB treatment) |
| **or** | **or** | **and** |  |
| *M. tuberculosis* culture positive sputum, and clinical findings or imaging indicative of gastrointestinal TB, and no other etiology identified for gastrointestinal findings | Weaker histopathological evidence (e.g. granulomata only) | Improvement in response to TB treatment |  |
| **or** | **and** |  |  |
| Strong histopathological evidence of TB (e.g. caseous necrosis plus AFBs) | Improvement in response to TB treatment |  |  |
| **or** |  |  |  |
| *M. tuberculosis* PCR/GeneXpert MTB ® positive |  |  |  |

**Supplementary figure 1: Recruitment and classification of study participants**

88

sequential suspected GI TB patients

11

Unable to get consent

77

patients included in dataset

8

Unknown final diagnosis

69

patients included in analysis

25 confirmed TB

13 probable TB

14

possible

TB

52

GI TB cases

17

non-TB diagnosis

12 malignancy

2

other infection

3

other diagnosis

**Supplementary table 3: Mean blood test results during initial diagnostic work-up**

|  | **Confirmed & probable TB cases** | **Non-TB cases** | ***p* value for difference in mean** |
| --- | --- | --- | --- |
| Hemoglobin (g/dL) | 10.80 | 10.35 | 0.64 |
| White blood cells (x10^9^/L) | 7.95 | 9.58 | 0.34 |
| Platelets (x10^9^/L) | 346.81 | 288.99 | 0.34 |
| Lymphocytes (%) | 18.89 | 15.73 | 0.39 |
| Creatinine (umol/L) | 109.24 | 152.79 | 0.56 |
| Glucose (mmol/L) | 6.54 | 7.33 | 0.30 |
| Albumin (g/L) | 26.17 | 21.54 | 0.07 |
| Bilirubin (mmol/L) | 13.35 | 24.04 | 0.18 |
| ALT (U/L) | 44.68 | 29.69 | 0.48 |
| AST (U/L) | 38.97 | 31.54 | 0.54 |
| ALP (U/L) | 172.96 | 201.48 | 0.73 |
| LDH (uL) | 266.71 | 208.75 | 0.23 |
| Prothrombin time (seconds) | 14.24 | 14.29 | 0.92 |

**Supplementary table 4: Gastrointestinal biopsy and ascitic fluid GeneXpert® MTB results by type and site**

| **Specimen site/type** | **Negative** | | **Positive** | | **Total** | |
| --- | --- | --- | --- | --- | --- | --- |
|  | **N** | **%** | **N** | **%** | **N** | **%** |
| Large bowel | 11 | 65 | 6 | 35 | 17 | 24 |
| Small bowel | 9 | 56 | 7 | 44 | 16 | 22 |
| Ascitic fluid | 15 | 100 | 0 | 0 | 15 | 21 |
| Peritoneum | 3 | 60 | 2 | 40 | 5 | 7 |
| Liver | 2 | 50 | 2 | 50 | 4 | 6 |
| Lymph node | 3 | 75 | 1 | 25 | 4 | 6 |
| Omentum | 1 | 50 | 1 | 50 | 2 | 3 |
| Other | 6 | 67 | 3 | 33 | 9 | 13 |
| **Total** | **50** |  | **22** |  | **72** |  |
